# Supplementary material for: The causal relationship between the human gut microbiota and pyogenic arthritis: a Mendelian randomization study
Source: Front Cell Infect Microbiol. 2024 Nov 26;14:1452480. doi: 10.3389/fcimb.2024.1452480 (PMC11629706; doi:10.3389/fcimb.2024.1452480)
Supplement: Supplementary file 1 [file DataSheet1.zip › mendelian test/ebi-a-GCST90027632.csv_forest.pdf]

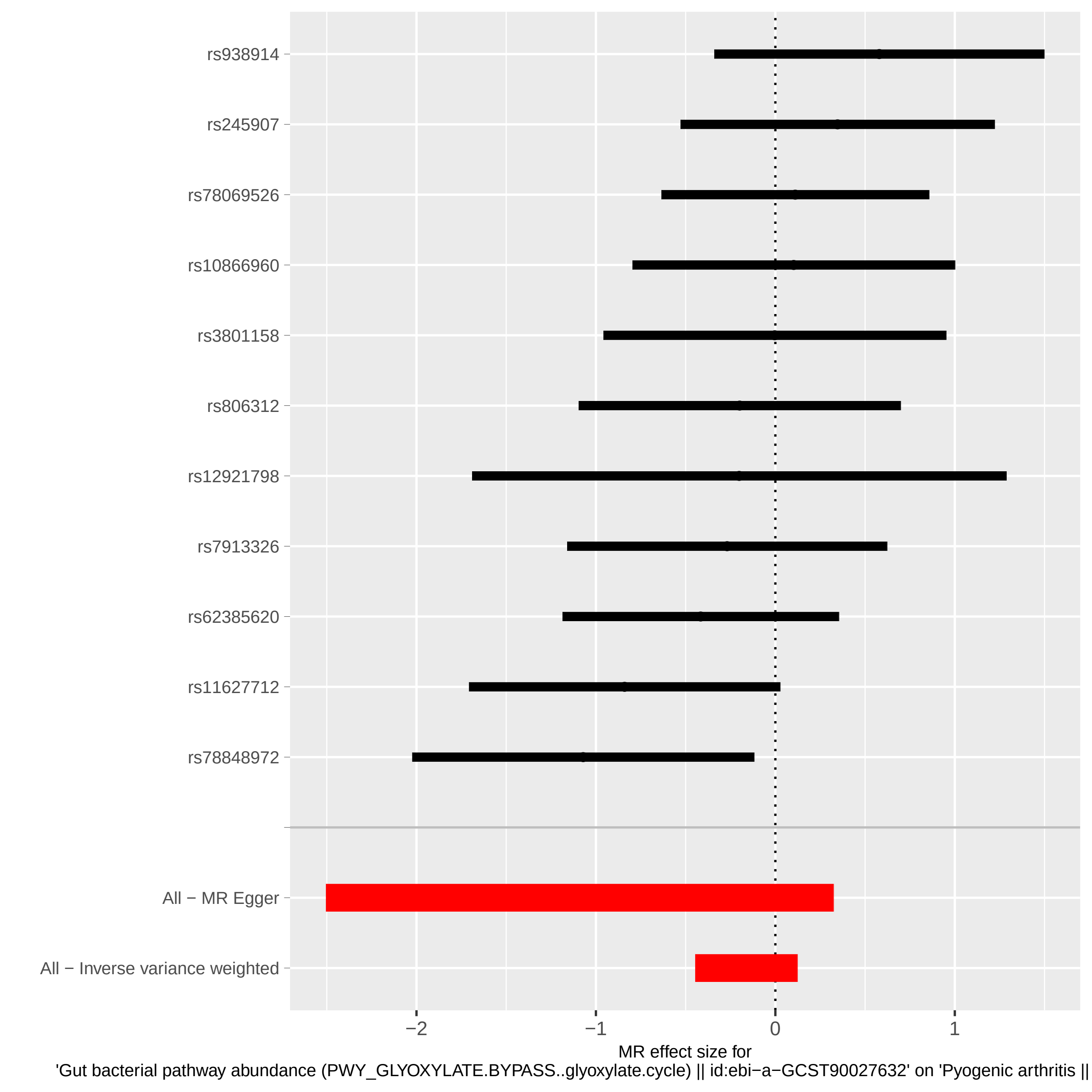

rs938914
rs245907
rs78069526
rs10866960
rs3801158
rs806312
rs12921798
rs7913326
rs62385620
rs11627712
rs78848972
All − MR Egger
All − Inverse variance weighted
−2
−1
0
1
MR effect size for
'Gut bacterial pathway abundance (PWY_GLYOXYLATE.BYPASS..glyoxylate.cycle) || id:ebi−a−GCST90027632' on 'Pyogenic arthritis ||
